# Palliative Care in Acute Heart Failure

**DOI:** 10.1007/s11897-020-00494-8

**Published:** 2020-10-29

**Authors:** James M. Beattie, Irene J. Higginson, Theresa A. McDonagh

**Affiliations:** 1 Cicely Saunders Institute of Policy and Rehabilitation, King’s College London, London, UK; 2 School of Cardiovascular Medicine and Sciences, King’s College London, London, UK

**Keywords:** Palliative care, Acute heart failure, Needs assessment, End-of-life-care

## Abstract

**Purpose of Review:**

Palliative care is increasingly acknowledged as beneficial in supporting patients and families affected by heart failure, but policy documents have generally focused on the chronic form of this disease. We examined palliative care provision for those with acute heart failure, based on the recently updated National Consensus Project Clinical Practice Guidelines for Quality Palliative Care.

**Recent Findings:**

The commonest reason for hospitalization in those > 65 years, acute heart failure admissions delineate crisis points on the unpredictable disease trajectory. Palliative care is underutilized, often perceived as limited to end-of-life care rather than determined by regular systematic needs assessment. No dominant paradigm of palliative care provision has emerged from the nascent evidence base related to this clinical cohort, underscoring the need for further research.

**Summary:**

Embedding palliative support as mainstream to heart failure care from the point of diagnosis may better ensure treatment strategies for those admitted with acute heart failure remain consistent with patients’ preferences and values.

## Introduction

Heart failure (HF) is a modern epidemic affecting close to 40 million people worldwide. The Rotterdam study estimated the prevalence to be about 2% in the general population, rising to 17.4% in those aged ≥ 85 years [1]. Linked to societal aging and also improved treatments for a range of cardiovascular diseases, projections have suggested a 46% increase in the prevalence of HF between 2012 and 2030 [2]. The incidence of HF is equivalent to the combined incidence of four common cancers—lung, breast, bowel, and prostate [3]—and the lifetime risk appears to be similar at 1 in 5 for both men and women. However, for men the incidence doubles during each decade between 65 and 85 years, while for women the incidence trebles over the same age deciles [4]. Heart failure is the commonest reason for hospital admission in those aged > 65 years, this inpatient care accounting for much of the expenditure required to treat this condition, the annual cost estimated to be at least US$108 billion for health economies globally [5].

Heart failure results from a diverse range of etiological cardiovascular conditions causing a spectrum of systolic and diastolic dysfunction, often mediated by disparate patterns of ventricular remodeling. The Heart Failure Association (HFA) of the European Society of Cardiology (ESC) has characterized three HF phenotypes based on assessment of left ventricular ejection fraction (EF) [6]: HF with a reduced EF (HFrEF), when the EF is < 40%, HF with a mid-range or mildly reduced EF (HFmrEF) [EF 40–49%], and HF with preserved EF (HFpEF) [EF ≥ 50%] respectively. The clinical presentation is similar across this HF phenotypic spectrum, the diagnosis usually considered in people presenting with effort dyspnea or orthopnea, easy fatiguability, and exhibiting a collection of stereotypical clinical features.

Heart failure can develop insidiously, or other patients present in an emergent fashion with de novo acute HF (AHF), when rapidly progressive symptoms and signs develop over a short period. AHF accounts for only about one-third of all HF admissions, the majority associated with acute decompensation of chronic HF (ADCHF) [7], and it has been argued that these clinical presentations should be regarded as distinct clinical entities. In a recent study of 370 consecutive patients hospitalized with dyspnea, echocardiographic evidence of left ventricular dysfunction, and elevated NT-proBNP, ADCHF was diagnosed in 80%, 62%, and 28% of those with HFrEF, HFmrEF, and HFpEF respectively [8].

It is beyond the scope of this review to describe the treatment of acute HF in detail, but as outlined in recent clinical guidelines, the required pharmacologic approaches and other measures are defined by congestion and hypoperfusion profiling (warm and wet or cold and dry) [6, 9]. Congestion demands the use of loop diuretics by a variety of dosing regimens. Other patients require vasodilator, inotropic or vasopressor therapy, these latter treatments not without risk. Increasingly, further intervention includes non-invasive ventilation, while intubation for mechanical ventilation, the use of extracorporeal membrane oxygenation (ECMO) or mechanical circulatory support (MCS), necessitates admission to a cardiovascular intensive care unit (ICU). Close monitoring is required, and a recent American College of Cardiology expert consensus document elegantly describes risk assessment and decision making relevant to the management of such patients [10•]. An inadequate response to therapy or in-hospital worsening of HF auger a poor prognosis [11]. Based on the National Heart Failure Audit (NHFA) data for 68,266 adults admitted with HF to hospitals in England and Wales between April 2017 and March 2018, 66% with HFrEF, the inpatient mortality was 10.1% [12]. In EuroHeart Failure Survey II, the in-hospital mortality was greater for AHF at 8.1% compared to 5.8% for ADCHF [13]. In contrast, a non-fatal admission with ADCHF portends worse intermediate and long-term outcomes. These patients have a longer duration of documented HF and are often older with more comorbidities. Earlier NHFA data suggested a 1-year all-cause mortality of 56% for those aged > 75 years [14].

Depending on the underlying etiology, appropriately treated acute ventricular dysfunction may recover in so-called myocardial remission, but the majority surviving the index acute phase go on to develop chronic HF. Thereafter, the clinical course is similar to other life-limiting diseases such as chronic obstructive pulmonary disease (COPD) or renal failure, typically manifest in a roller coaster disease trajectory with a pattern of gradual decline, interspersed with inflection points marking ever more frequent acute clinical crises. These include those related to ADCHF, sometimes evident in a sentinel cluster of hospital admissions close to the end of life [15]. Cardiovascular causes of death predominate, and while lethal arrhythmia as a mode of dying is diminishing, this still accounts for 17% of all fatalities within 30 days of an admission for ADCHF [16, 17]. Heart failure patients surviving 3–5 years out from their initial diagnosis increasingly die from unrelated comorbidities such as cancer, and indeed various serological factors directly linked to the HF state may be implicated in tumorigenesis [18].

## Palliative Care and Heart Failure

Evolving from the hospice movement of the 1960s and 1970s, palliative care (PC) is regarded as an essential component of universal health coverage. The most widely accepted definition is from the World Health Organisation (WHO) which in 2002 [19] described this as:

*..an approach that improves the quality of life of patients and their families facing the problems associated with life-threatening illness, through the prevention and relief of suffering by means of early identification and impeccable assessment and treatment of pain and other problems, physical, psychosocial and spiritual.*

Originally closely aligned to oncologic practice, over the past 20 years or so, with increased awareness that the PC needs of those living with HF or cancer are similar [20, 21], HF has been in the vanguard of extending this support beyond that founding base. Palliative care is now incorporated within national and international HF treatment guidelines [6, 22], and has been the subject of recent position statements by the ESC HFA and the European Association of Palliative Care (EAPC) [23••, 24••].

To some extent, the features of PC as described by the WHO are central tenets of all clinical practice, and essentially any clinician should be able to offer so-called generalist or primary PC in providing basic symptom control, elucidating goals of care, and enabling clinical coordination. Indeed, a combination of primary and specialist PC has been posited as a potential sustainable model of HF PC service provision [25]. However, the skillset of PC specialists facilitates more comprehensive and effective targeting of complex physical symptoms and the psychological or existential distress commonly affecting those with this significant disease, their care needs often beyond the tacit knowledge and experience of HF professionals. Good communication is central to the practice of PC, and integrating such professionals within a multidisciplinary team (MDT) fosters shared decision making to better ensure that established or proposed elements of guideline-directed medical therapy (GDMT) remain consistent with patients’ and families’ goals of care, preferences, and values, often subject to change in the face of disease progression.

It is important to emphasize that including PC in the treatment strategy from the initial diagnosis of HF is not at odds with the co-provision of life-prolonging therapies from that point and throughout the disease trajectory, this professional collaboration continuing beyond the death of patients in affording supportive care to their bereaved relatives or other close persons [26••]. Thus, PC should not be regarded as limited to end-of-life care which constitutes just one of eight domains defining core standards of clinical practice by the National Consensus Project for Quality Palliative Care [27] (Table 1). We shall base this review of PC for acute HF on this suite of quality domains.

**Table 1:** Key domains for quality palliative care

Structure and processes of care provision	
Addressing physical symptoms	
Psychological and psychiatric care	
Social care	
Spiritual, religious, and existential support	
Culturally competent care delivery	
Care near the end of life	
Ethical and legal constructs relevant to care	

Based on the National Consensus Project Clinical Practice Guidelines for Quality Palliative Care, 4th Edition. (Modified from Ferrell et al. [27])

## Structure and Processes of Care Provision

Integrating PC within HF services depends on the configuration of national and local healthcare structures and is contingent on the organizational frameworks underpinning clinical care, including regulatory systems defining care eligibility and reimbursement. There is wide disparity internationally [28]. For example, in the United States (US) as of 2018, 50% of hospitals with ≥ 300 beds offered specialist PC consultations to adult inpatients, a service less accessible in Europe, although 8 European countries have established fully integrated HF PC services [29, 30]. On the African continent where the prevalence of HF is rising, this constitutes the commonest cardiovascular condition requiring hospitalization. While some African countries have adopted national PC policies, even when such services do exist, resources are limited, with scant data on HF-related service structures [31].

As the applicability of PC for HF has gained traction in recent years, hospital admission has been specifically cited as an opportunity to integrate this form of support [32], but incorporating this as standard care remains difficult, particularly for those with acute HF syndromes [10•]. Palliative care involvement during unplanned hospitalizations is exceptional, and in North America, this has been documented at only 3.9%, even in those patients requiring mechanical ventilation [33, 34]. For England and Wales, NHFA data have demonstrated that only 3.1% of acute HF patients were referred to PC at their index admission, this rising to 7.3% following readmission [35]. Similarly, registry data from Sweden recorded that just 4.2% of HF decedents received such support during their last week of life [36].

Many factors contribute to this relative lack of PC engagement [37]. The landscape of cardiovascular clinical practice is dominated by the rule of rescue in preserving life at all costs, prompting a sense that the need to consider PC implies professional failure. Cardiology professionals often shy away from opening conversations to explore treatment preferences and the applicability of PC in the face of clinical decline, concerned this discourse might undermine patients’ and families’ trust, and dash their hopes for the future [38]. A misconception still prevails that PC is limited to end-of-life care.

Despite the uncertainty intrinsic to the HF disease trajectory, prognostication has often dominated consideration for PC involvement. As with chronic HF, multivariate risk scores for acute HF populations have also been developed [39], but prognostication for individual patients is still challenging. The surprise question (“Would you be surprised if this patient were to die over the next year?”) has been shown to have a sensitivity and specificity of 85% and 59% respectively for acute HF inpatients when used by HF specialists, but performance declines with less experienced staff, and judgment based on this approach remains largely intuitive [40]. Although some clinical descriptors of advanced (Stage D) HF predict mortality risk, these show poor correlation with symptomatic status, and we would argue that the requirement for PC support should be judged on the basis of systematic needs assessment rather than prognosis [41].

Patient-reported outcome measures (PROMs) characterize the experience of those living with conditions such as HF, enabling clinicians to be attentive to the often-nuanced needs of each patient, and to assess changes over time or following interventions. Their use facilitates person-centered care. To be effective, PROMs must exhibit robust psychometric properties, demanding rigorous verification of reliability and reproducibility, validity, responsiveness, and acceptability. The most widely adopted HF-specific PROMs are the self-administered 21-item Minnesota Living with Heart Failure Questionnaire (MLHFQ) and the 23-item Kansas City Cardiomyopathy Questionnaire (KCCQ). The MLHFQ and KCCQ incorporate multiple domains using Likert scales to quantify the presence and severity of physical symptoms, and any psychosocial impact. Other instruments are more geared to evaluate PC needs. Based on estimating symptom burden, the 10-item Edmonton Symptom Assessment System (ESAS) also uses numeric rating and has been subject to significant modification since first developed in 1991 [42]. The Functional Assessment of Chronic Illness Therapy–Palliative Care (FACIT-Pal) Scale is a 46-item self-reported measure appraising physical, social, emotional, and functional well-being, and incorporating a 19-item PC subscale assessing symptoms, social interaction, and meaning in life [43]. The 10-item Integrated Palliative care Outcome Scale (IPOS), which is freely available (www.pos-pal.org), has also been demonstrated as feasible and acceptable in the comprehensive assessment of HF symptoms, and to be deliverable by HF nurse specialists after appropriate training [44•]. Developed in Australia, a HF-specific instrument, the Needs Assessment Tool: Progressive Disease–Heart Failure (NAT: PD-HF) aids assessment of the physical and psychosocial well-being of both HF patients and their informal carers across a range of care settings, defining thresholds of concern useful in delineating those in need of specialist involvement [45•]. Both the IPOS and NAT: PD-HF instruments have been utilized in those admitted with acute HF [46, 47].

Most randomized clinical trials (RCTs) of HF PC have been carried out in North America and Northern or Western Europe, much of this work focused on chronic HF. While the evidence base remains relatively light across an array of interventions subject to various assessment methodologies, recent meta-analyses suggest that this support improves HF patients’ quality of life (QoL) and reduces their symptom burden, with no adverse effect on mortality [48•, 49••]. The reported impact on rehospitalization rates is mixed, this varying between a neutral effect or significant reduction.

Hospital-based PC teams likely improve care for those affected by a variety of underlying diagnoses, and particularly relevant to this review, several studies have purposely examined the effects of a PC intervention for people admitted with acute HF. Based in a tertiary-care urban hospital setting, Sidebottom and colleagues compared outcomes in 232 patients randomized to receive standard care with or without an initial inpatient PC review, followed by additional consultations at 1 and 3 months post-discharge [50]. Patients randomized to the PC treatment arm had greater alleviation of symptoms as evaluated by ESAS and the Patient Health Questionnaire-9, with increased participation in advance care planning (ACP), and no significant effect on hospice use, 30-day readmission rates, or mortality.

Hopp et al. studied 85 urban-living patients, predominantly African Americans, admitted with ADCHF, and randomized to a PC consultation in addition to usual care [51]. There were no significant inter-group differences for the primary endpoints in choosing a “Do not attempt cardiopulmonary resuscitation” (DNACPR) policy during the index or later hospitalizations, or their preferences for comfort care, including the use of hospice post-discharge. Of this study cohort, 23.8% died within 3–6 months of randomization and the PC intervention did not influence survival.

The landmark Palliative Care in Heart Failure (PAL-HF) trial was a single-center unblinded RCT undertaken at Duke University Hospital in Durham, NC [52••]. This involved HF inpatients recruited within 48 h of planned discharge (*n* = 148) or within 2 weeks of hospital discharge (*n* = 2), deemed to be at high risk of rehospitalization or death. The primary study objective, coordinated by a PC nurse practitioner, was to examine the impact on health-related QoL of a supplementary multi-component PC intervention, delivered by an MDT after discharge, in addition to usual care. As outlined in the trial protocol, the PC intervention targeted symptom relief, spiritual concerns, and ACP. Over a 6-month period post-discharge, patients randomized to the PC group had significant improvements in KCCQ and FACIT-Pal scores compared to those receiving usual care, these benefits more obvious in men. Positive effects on secondary endpoints included better spiritual well-being and less anxiety/depression. There were no demonstrable effects on rehospitalization rates or mortality. In PAL-HF, the benefits of PC continued throughout the observational period of the study; however, other reports suggest early gains evident after initiating inpatient PC support might wane over time [53].

Wong and colleagues compared outcomes of a transitional PC model on 84 recently discharged HFrEF patients, judged by their clinicians to be in the last year of life [54]. In this multi-site RCT based in Hong Kong, following pre-discharge assessment, PC nurse case managers, supported by a PC physician within an MDT process, undertook weekly home visits/telephone consultations over a 4-week period, followed by regular contacts through 12-months follow-up. For the intervention group, the primary endpoint of readmission rate was significantly reduced. They also showed improved secondary outcomes with reduced symptom intensity as measured by ESAS, and improved QoL when assessed by validated Chinese versions of the Chronic Heart Failure and McGill Quality of Life Questionnaires.

The Social Worker-Aided Palliative care intervention in high-risk patients with Heart Failure (SWAP-HF) is a recent prospective RCT conducted at the Brigham and Women’s Hospital in Boston, MA [55]. This study involved 50 high-risk ADCHF patients recruited during admission or shortly thereafter, randomized to intervention or usual care. Over a 6-month period post-discharge, a PC-trained social worker led conversations with the intervention group, evaluating their symptom burden, QoL, patients’ and families’ understanding of prognosis, and their preferences for end-of-life care, coordinating review by a PC physician as necessary. A higher proportion of the PC group had more realistic understanding of their prognoses, these better aligned with their physicians’ opinions, and there was more documentation of ACP and end of life treatment preferences compared to those receiving usual care, this information readily accessible to non-study staff in patients’ clinical charts. There were no adverse outcomes in relation to depression/anxiety, spiritual distress, or worsening KCCQ scores.

Insofar as the above RCTs appear to show a relative uniformity in positive outcomes across the spectrum of PC interventions offered to these acute HF cohorts, we must add a caveat that, in the main, usual care was not systematically described in these studies, and may have been heterogeneous both within single centers and between sites. Palliative care interventions are relatively complex, and recent work has proposed a means of classification of usual care which might better assure the validity of perceived outcomes [56].

As apparent in the studies described above, no dominant model of the integration of PC support for HF inpatients has emerged. Hospital-based PC services might be provided using on-site beds assigned to PC, but perhaps more commonly, this care element is made available to those occupying ICU, acute, or sub-acute beds through an advisory or consultative in-reach service involving specialist PC physicians or nurses.

Collaborative MDT working is considered central to the co-management required of effective HF PC provision. The MDT membership will vary between organizations, and the array, weighting, and means of delivery of specific care constituents will also be determined by individual patients’ needs in relation to their heart disease, comorbidities, sociocultural characteristics, and health literacy. Productive MDT activity requires agile and responsive processes, and within this construct, it is clearly important to undertake task allocation and define the responsibilities of team members. It may be helpful to designate one individual to act as interlocutor, liaising with the patient, family, and any contributing external agencies to foster good communication and care coordination between acute, ambulatory, and community-based services. Transitions between treatment settings present particular risks of care fragmentation [57]. It is important to ensure continuity of care with robust systems of information transfer, and the ready availability of professional advice and access to HF therapies for patients discharged from hospital to their own home or care home, to a skilled nursing facility, or a hospice.

## Addressing Physical Symptoms

Optimal GDMT improves longevity, and those adhering to this may also experience symptomatic benefit. However, for many HF patients now living longer with this progressive condition, survivorship becomes manifest in a fairly liminal existence, burdened by a constellation of refractory symptoms, comprehensively described elsewhere [58•]. Indeed, based on the Evaluation Study of Congestive Heart Failure and Pulmonary Artery Catheter Effectiveness (ESCAPE) sub-study, following hospitalization with ADCHF, some patients might be willing to trade-off days alive for a better QoL [59]. The spectrum and intensity of the symptom burden of those with severe HF are shown in Table 2. We shall address some of these symptoms in detail.

**Table 2:** Refractory symptoms in severe heart failure

Symptom	Prevalence (%)	Intensity (0–10)
Malaise	100	5.1
Dyspnea	92	5.3
Tiredness	92	5.6
Pain	60	2.8
Anorexia	82	3.7
Nausea	28	1.0
Anxiety	68	3.2
Depression	66	3.0
Drowsiness	6	3.6

Spectrum and intensity (mean ESAS score) of refractory symptoms–NHYA III/IV HF

*NYHA*, New York Heart Association; *ESAS*, Edmonton Symptom Assessment System (modified from O’Leary et al. [20])

### Dyspnea

Dyspnea is common in HF and may vary in duration and intensity in response to exertion, emotion, or environmental triggers. Those with advanced chronic HF become breathless on minimal exertion such as those required of the activities of daily living (ADL). Breathlessness as described by patients with a variety of advanced disease states, including HF, may also be characterized as acute, episodic, or continuous [60]. Based on the Charité Emergency Medicine (CHARITEM) study in Germany, acute dyspnea was the main complaint in 65.1% of those requiring emergency hospital admission with HF [61], and although pulmonary edema as assessed by clinical examination or lung ultrasound is common in such patients, breathlessness at rest may not be their dominant symptom. In a retrospective review of 311 acute admissions to an academic HF service in England, only 42% reported breathlessness at rest, the others becoming breathless on slight exertion similar to ambulatory patients with severe disease living in the community [62]. At presentation with acute HF, the severity of dyspnea correlates with the degree of congestion and requires the use of parenteral loop diuretic therapy with careful monitoring of renal function. Those with a history of paroxysmal nocturnal dyspnea or orthopnea, when acute pulmonary edema is more common, require to be nursed in a supported upright or semi-upright (Fowler) position. Opiates have demonstrable efficacy in those with chronic breathlessness, but most data are based on patients with COPD. However, the recently published phase III BreatheMOR-HF RCT of the daily administration of a 20 mg modified release morphine preparation shows benefit in those with chronic HF [63]. Opiates may also alleviate the anxiety and distress associated with acute breathlessness; however, the safety of the previously widely endorsed use of short-term intravenous opiates in those with acute pulmonary edema has been questioned of late [64], and the outcome of the ongoing MIdazolam versus MOrphine in Acute Pulmonary Edema (MIMO) Trial (ClinicalTrials.gov Identifier: NCT02856698) remains to be determined. In those with demonstrable hypoxemia, supplemental oxygen therapy is indicated, the need for non-invasive or mechanical ventilation being considered if this persists or there is evidence of respiratory acidosis. It is important to ensure that such treatment escalation is consistent with patient preferences for care.

### Fatigue

Alongside breathlessness, fatigue is an almost universal symptom in HF, this being documented in 95% of those affected [65]. Fatigue was specifically recorded in 39% of patients presenting with incident HF and cited as their worst symptom by 32% of 371 patients admitted with ADCHF, this responding less well to treatment than dyspnea [66, 67]. The background is likely multifactorial, and a variety of pathophysiologic mechanisms may contribute including low cardiac output with reduced tissue perfusion, structural and metabolic abnormalities of the myocardium and skeletal muscle, and autonomic dysfunction [68, 69]. Patients with fatigue are usually older, female, and with multiple comorbidities including depression and frailty-cachexia syndromes. There is a particular association with HF hospitalization, but fatigue per se is not a strong predictor of cardiovascular or all-cause mortality [65, 67]. However, this relatively refractory symptom impacts patients’ functional capacity and psychosocial well-being, often resulting in them becoming more dependent on others to fulfill required ADLs.

### Pain

Cicely Saunders was the first to conceptualize the theory of total pain as comprising physical, spiritual, psychological, and social constituents, and this has been proposed as a model relevant to those with advanced HF [70]. While the impact of pain is often unrecognized in this condition, the multi-site PAIN-HF study conducted across the US demonstrated that 84% of ambulatory patients with severe chronic HF experienced significant pain affecting QoL [71]. A comparable prevalence of pain at 85% was reported for acute HF patients assessed during hospitalization in Norway [72]. In both these studies, most patients exhibited HFrEF and pain may be more common in ADCHF patients with this phenotype [73]. This symptom appears to be relatively refractory, and 69% of 169 acute HF inpatients in Canada still complained of moderate to severe pain when assessed at the point of hospital discharge, this continuing and of similar intensity in 41% when reassessed 6 weeks later [74]. Pain experienced by HF patients is not usually cardiogenic, and a previous review has comprehensively described the pathophysiologic mechanisms and outlined treatment strategies [75]. As with breathlessness, opiates appear to be relatively effective and non-steroidal anti-inflammatory preparations are generally contraindicated.

## Psychological and Psychiatric Care

Importantly, symptoms do not occur in isolation but rather in clusters, and there is often an interdependency between those of somatic and psychologic origin [76]. Anxiety may heighten the perception of breathlessness, and as outlined above, fatigue may be associated with depression.

### Anxiety

The reported prevalence of anxiety in HF varies due to disparities in methods of assessment, but a recent meta-analysis reported a random effects pooled prevalence of 13% for a formal anxiety disorder, 29% for probable clinically significant anxiety, and 56% for heightened symptoms of anxiety [77]. Based on the Hospital Anxiety Depression Scale, moderate to severe anxiety was documented in 18% of patients hospitalized with HF [78], this correlating with increased rates of rehospitalization and mortality, such outcomes less frequently reported for those with chronic HF [79].

### Depression

Clinically significant depression affects about 22% of HF patients, and unsurprisingly, prevalence varies with functional limitation, documented as 11% and 42% for New York Heart Association classes I and IV respectively [80]. Compared to anxiety, depression has been more consistently linked to poorer survivorship, and associated with a twofold increase in mortality for the general HF population. However, for those with acute HF, the risk may be greater. In the OPERA-HF observational study of HF admissions, 15% of 242 patients with moderate to severe depression had a near fivefold increase in all-cause mortality by 12 months post-discharge [81].

The background to these adverse outcomes for anxiety and depression is unclear and may relate to both altered biologic mechanisms involving increased inflammation and platelet aggregability with endothelial dysfunction, or the adoption of adverse health behaviors such as poor medication adherence or physical inactivity [82•]. Treating anxiety and depression may be based on psychotherapeutic, educational, or pharmacologic approaches. For all treatment modes, evidence has largely evolved from those with chronic HF. Cognitive behavioral therapy has shown some benefits for both anxiety and depression. Medication in the form of selective serotonin reuptake inhibitors has generally been regarded as first-line therapy based on their relative safety and effectiveness in non-cardiac patients, but results vary in those with HF. Two RCTs examining the effects of sertraline (SADHART-CHF) and escitalopram (MOOD-HF) showed responses equivalent to placebo [83, 84]. Other drugs such as tricyclic antidepressants may be pro-arrhythmic, and venlafaxine and duloxetine may worsen HF, and are best avoided. The α-2 antagonist mirtazapine is commonly prescribed for depression; however, the efficacy and safety of this drug has not been systematically evaluated in the HF population. Accessing psychiatric expertise through the MDT process may facilitate optimal treatment and improve clinical outlook.

### Cognitive Impairment

Disorders of cognition are common in those with HF and may occur as both acute and chronic progressive forms. Delirium, an acute confusional state evident as inattention and global cognitive dysfunction, has been reported in 17–35% of patients admitted with de novo AHF or ADCHF, and associated with poor outcomes in worsening HF during hospitalization, increased length of stay and readmission rates, and greater short and long-term cardiovascular and all-cause mortality [85, 86]. Delirium may be directly related to acute HF in hypoxia or hypotension-related cerebral hypoperfusion. As with patients admitted with other acute medical conditions, delirium may be triggered by exposure to unfamiliar, apparently threatening surroundings, compounded by disruption of sleep architecture. Management includes reassurance and reorientation in space and time. Significant agitation may respond to haloperidol, with monitoring for QT prolongation, or the use of other anti-psychotic agents such as risperidone. Benzodiazepines may be particularly helpful in delirium due to alcohol withdrawal.

Chronic cognitive impairment is estimated to affect about 40% of HF patients, irrespective of ejection fraction phenotype [87]. This can vary in severity across several domains including attention, memory, speech and language processing, learning, and executive function. A previous study found that 80% of 774 patients admitted with ADCHF were impaired in at least one domain, this predominantly involving memory, processing speed, and executive function [88]. Depressed patients were twice as likely to be impaired in all three of these domains. No causal link has been established with HF although cerebral hypoperfusion and occult cardioembolic disease have been proposed as potential mechanisms. Impaired cognition may also result from specific forms of dementia across a range of pathophysiologic processes, these conditions simply co-existing in the typically affected elderly population. To date there is no evidence to suggest that any drugs within GDMT for HF directly affect cognition. However, downregulation of the enzyme neprilysin increases cerebral deposition of amyloid-β protein, a hallmark of Alzheimer’s disease. Following the positive outcomes of the PARAGON-HF study in those with HFrEF, the angiotensin receptor–neprilysin inhibitor sacubitril/valsartan is increasingly prescribed. A retrospective analysis of the PARAGON-HF study group revealed no excess of dementia compared to other HF trial populations [89], but a prospective study is currently underway to assess the effects of neprilysin inhibition on cognitive function in those with chronic HF (NCT02884206). The EAPC has issued a white paper describing optimal PC for those with dementia [90]. The high prevalence of cognitive impairment in chronic HF suggests that enactment of advance directives might be useful in those at risk of losing capacity, but such constructs have been poorly espoused by this population, being documented in only 13% of those admitted with ADCHF [91].

## Social Care

Socioeconomic disadvantage is widely acknowledged as contributing to the risk profile for cardiovascular disease, often evident in a final common pathway as HF. A recent American Heart Association (AHA) Scientific Statement specifically addressing the social determinants of health (SDOH) for this clinical cohort reiterated the WHO definition of these as “the circumstances in which people are born, grow up, live, work and age…..and the systems put in place to offer health care and services to a community” [92••]. Against this multifaceted backdrop, people with HF who are of non-white race or otherwise marginalized, those with modest educational attainment, economic instability, poor living conditions, or lacking in social support or access to effective health care structures, face worse outcomes with this condition. This vulnerability was evident in a study of 690 Medicare beneficiaries > 65 years discharged alive after HF hospitalization who showed a close to threefold increase in 90-day mortality if they were exposed to only one of nine possible SDOH as defined in the REGARDS (Reasons for Geographic and Racial Differences in Stroke) study, even when patients discharged to hospice were excluded [93]. The AHA statement explicitly highlighted poor access to PC in such disadvantaged populations, and a systematic review has demonstrated that people living in high-income countries with low socioeconomic standing experience poorer quality end-of-life care with increased use of acute medical services in the 3-month period before death, higher rates of death in hospital, and lesser involvement of specialist PC services during their last year of life [94].

Socioeconomic deficits affect the well-being of both HF patients and their informal carers, the latter often sharing a common heritage, lived environment, and also mediating health behaviors. There is also a gendering issue in that for men, a disproportionate amount of that caring burden is assumed by women, whereas older women, not infrequently affected by HF, may be bereft of spousal support. Clearly such risk elements are multifactorial, inter-related, and cumulative, and an intersectional approach has recently been proposed as appropriate to the investigation of HF patient-carer dyads [95]. Sociodemographic subsets such as single person households, minority language groups, and those living in rural areas may be particularly exposed. Some of the factors underlying SDOH are not modifiable, but greater social work involvement and multiagency supportive care beyond the biomedical paradigm of clinical PC, might be beneficial in addressing the unmet needs of these vulnerable populations.

## Spiritual, Religious, and Existential Support

Spirituality is intrinsic to human nature, and supporting this has been proposed as central to the holistic bio-psychosocial-spiritual model required of PC for those with significant illness or near the end of life [96]. It is important to emphasize that spirituality is not synonymous with organized religion or religiosity, but rather a broader concept involving a sense of life’s purpose, meaning, connectedness, and transcendence to a higher power, nature, or humanity in toto.

Following the development of an international consensus in 2014, the EAPC defined spirituality as:

*The dynamic dimension of human life that relates to the way persons (individual and community) experience, express and / or seek meaning, purpose and transcendence, and the way they connect to the moment, to self, to others, to nature, to the significant and / or the sacred.*

To some extent, the above definition also encompasses existential well-being, the means by which people adjust to self, their community and environment, their inter-connectedness with others, and their degree of reconciliation with earlier life events [97]. Recent work emphasizes the inherent uniqueness of the spiritual needs of patient/carer dyads, and also their willingness to engage with professionals to foster resilience and coping skills arising from spiritual support [98]. For those active in religious observance, this might be provided through chaplaincy services, with secular pastoral care available to others. This study also suggested that patients and families would prefer all healthcare professionals to contribute to this element of care at some level, at least in engaging in empathetic patient-centered communication, and being what has been described as a “compassionate presence” [99]. Yet it is widely acknowledged that clinicians often feel ill-equipped to explore and respond to spiritual issues, highlighting the need for professional education in this field.

Spiritual well-being of HF patients tends to follow the vagaries of their physical and psychological health along the disease trajectory, and this has been specifically linked to acute hospitalization [100]. While there has been increased interest in addressing the spiritual needs of those with HF, and various psychotherapeutic approaches including life review have been proposed, a particularly effective model of support is yet to be elaborated [101].

## Culturally Competent Care Provision

National populations across the world are increasingly culturally diverse, this pluralism resulting from escalating global migration, the United Nations estimating that in 2015, more than 244 million people were living in a country other than that of their birth [102]. As clinicians we interact with patients and families who align with many cultures, culture being defined as “a system of shared ideas and meanings that underlie, influence and structure the ways in which people think and act in practical situations” [103]. Culture has a degree of granularity, and even across ethnocultural groups and within families, attitudes of individuals vary depending on how deeply rooted they are within their own culture, or their degree of acculturation with the dominant indigenous population within which they are embedded. Culture influences a person’s worldview and affects responses to significant illness, some searching for a sense of purpose in suffering coupled to their spiritual connection or faith tradition, this sometimes shaping treatment preferences and engagement with care processes and ultimately determining outcomes [104]. Open awareness of dying by those facing the inexorable progress of diseases such as HF is a philosophy supported by PC professionals, allowing people to come to terms with their situation, and as a means of developing care plans to better ensure a good death. However, such truth telling policies have emerged in predominantly Anglophone regions, and run counter to the sociocultural norms of other societies wherein death taboos exist in the belief that even discussing the dying process or imminent death will hasten that end. Preferences for non-disclosure may affect patient autonomy and undermine the principle of shared decision making. In some cultures, patients prefer to rely on collective decisions made within families, or to defer treatment choices to their physicians whom they regard as vested with epistemic authority. In such situations it is important that clinicians are aware of their own biases which might influence judgment calls. While the subtleties required of sensitive communication might be lost in translation if professionals and patient/carer dyads do not share a common first language, calibrating such conversations with the help of interpreters might engender trust. This may facilitate consensus building in taking forward clinically appropriate decisions regarding initiation of PC, resuscitation policies, and ACP for HF management. Health care providers need to have processes in place to support the range of cultural perspectives and needs specific to their local communities with cardiovascular disease [105•].

## Care Near the End of Life

Given the stuttering nature of the HF disease - death trajectory, recognition of the dying phase is challenging. A recent study of clinical decision making at the end of life, which included 81 acute HF inpatients, suggested that such discernment was often instinctive and cloaked with uncertainty [106]. Indeed, acknowledging uncertainty may itself be a useful premise on which to base further discussion with the patient and family, and to re-explore their values, goals, and preferences for treatment options in the setting of progressive clinical deterioration [107]. A reluctance of professionals to broach issues of death and dying is understandable, but evidence suggests that recently hospitalized HF patients frequently reflect on these matters and are fearful about dying in hospital which is the usual outcome [108, 109]. Many patients would prefer to die in familiar surroundings at home [110], and while achieving the preferred place of death is regarded as a key performance indicator of good quality end-of-life care, it is important to ensure that such patients are properly supported. In a recent retrospective study of 3981 HF decedents from the Swedish Register of Palliative Care, of those dying at home or in nursing homes, 17.2% died alone, with no family or healthcare professionals in attendance [36].

Hospice in the form of a dedicated palliative care facility is underutilized in HF. In the US, “hospice” care is available at home through the Medicare Hospice Benefit Program for patients certified as having a likely life expectancy of ≤6 months, and who choose to forgo curative therapy. It has been estimated that close to one-third of American HF patients have subscribed to this scheme by the time of death [111]. Enrolment tends to occur late in the course of the disease but is associated with reduced use of acute medical services.

Optimizing care at the end of life requires re-examination of the utility of previously prescribed medication and any implanted device therapy, the benefits or burdens of invasive measures to support hydration or nutrition, and the appropriateness of intensive care. For those distressed with multiple symptoms, anticipatory prescribing of all potentially necessary medication should be considered [112•]. Comprehensive GDMT for HF entails polypharmacy, but much of this helps maintain ventricular function, and withdrawal risks symptomatic deterioration. However, some long-prescribed drugs such as statins may be rendered superfluous in this disease phase and deprescription may be reasonable [113]. Similarly, the efficacy of anticoagulation may approach clinical equipoise depending on the clinical indication, although changing from warfarin to a direct anticoagulant may reduce the treatment burden. Patients with advanced HF increasingly receive continuous inotropic therapy as a form of palliation [114]. While inotropes can be administered at home, this may be impractical for some, obliging them to stay in hospital, and this therapy may be disallowed in those transitioning to hospice care.

The use of implantable cardioverter-defibrillator (ICD) therapy for the primary or secondary prevention of sudden death as directed by clinical guidelines is rising exponentially. While there is unequivocal evidence of benefit in device-eligible patients, there are numerous reports of patients receiving distressing and purposeless shocks close to the end of life [115]. The possibility of later device deactivation should be introduced as part of the consent process prior to the primary implantation when bundling with value-based ACP may be advantageous [116], revisiting this issue along the disease trajectory after crises, or when considering generator replacement. Society guidelines provide protocols for ethically appropriate ICD deactivation, and it is important to ensure the ready availability of trained staff and equipment to facilitate this process [117]. Similarly, MCS in the use of durable ventricular assist devices (VADs) as a bridge to decision making, recovery or transplantation, or as destination therapy, is becoming more common [118]. Since 2013, the Centers for Medicare and Medicaid Services in the US have mandated PC involvement in this process, this requirement driving a broader inter-disciplinary collaboration between cardiology, cardiac surgery, and PC [119, 120]. Palliative care supports preparedness planning for HF patients and families considering MCS, as well as supporting them towards the end of life, or if active discontinuation of VAD therapy is required following major device-related complications [121]. Similar issues arise if ECMO support for acute HF is being withdrawn, although there is less experience with that technology at this time [122]. ECMO is often employed in a time-limited emergent fashion, and families may have little concept of the nature or implications of this form of treatment, underscoring the need for sensitivity and good communication.

## Ethical and Legal Constructs Relevant to Care

The ethical principles of autonomy, beneficence, non-maleficence, and justice are clearly relevant to those with presenting with acute HF, particularly late in the disease course. Autonomy mandates that the competent patient be sufficiently informed of the nature of their condition to enable them to accept or refuse proposed treatment elements consistent with their preferences and values, this shared decision-making policy endorsed by the AHA [15]. If HF patients lose capacity, informal carers often assume a surrogate role as decision makers. Disagreements may arise between families and health care professionals on treatment policies when the involvement of a clinical ethicist may be useful, and these individuals may also help formulate a consensus in defining ceilings of care and the withdrawal of therapies now considered non-beneficial. The benefits of ACP or any previous statements by the patient are clearly relevant here, their status subject to the nature of the instrument enacted and local legislation. Policies on DNACPR also fall within this compass, and it is important to note that there is much variation across jurisdictions in the chain of responsibility for such orders and their legality, often influenced by societal and cultural factors.

In compiling this paper, we take a neutral stance and do not offer guidance on assisted suicide or euthanasia, but it should be noted that physician-assisted dying is legal in many countries and territories internationally.

## Conclusion

As with chronic HF, providing PC to those with acute HF presents challenges and opportunities. Organizational barriers might come into play in that some systems of care may not sanction inclusion of PC within the HF care bundle, or the necessary workforce may be unavailable. More commonly, there is still a failure to appreciate that the concurrent provision of PC alongside GDMT aimed at life prolongation is not incongruous. For many patients admitted with acute HF, some of whom will die in hospital, there is often a tension in reconciling the “treatment imperative” and the “ethical imperative” in the need to provide comfort care. This dilemma is evident in considering the clinical characteristics behind the I-NEED-HELP mnemonic [123], proposed as an aide-mémoire describing those potentially requiring advanced HF therapies, but which are no less applicable as markers of PC need. Embedding PC within a HF MDT from the time of diagnosis avoids silo working, needs assessment undertaken as an iterative process along the capricious disease trajectory enabling incremental PC involvement during disease progression, with formulation of appropriate treatment escalation and withdrawal policies as patients decline towards the end of life. Close to death, it is important that HF professionals trusted by patients and families remain engaged, avoiding a perception of abandonment.

Themes emerging from a recent review examining the barriers and facilitators of a “good death” in HF [124] included the following: (a) effective communication between patients/families/healthcare providers; (b) good clinical navigation through the terminal phase and dying process; (c) the avoidance of futile invasive interventions; (d) good symptom control; (e) timely access to specialist PC, including bereavement care; and (f) achieving the preferred place of care and death. The domains included in the National Consensus Project Clinical Practice Guidelines provide useful benchmarks of the quality of PC required for those with acute and chronic HF, up to and including that final transition.
